# Supplementary material for: Wing bone geometry reveals active flight in Archaeopteryx
Source: Nat Commun. 2018 Mar 13;9:923. doi: 10.1038/s41467-018-03296-8 (PMC5849612; doi:10.1038/s41467-018-03296-8)
Supplement: Supplementary file 1 — Supplementary Information [file 41467_2018_3296_MOESM1_ESM.pdf]

Supplementary Information accompanying "Wing bone geometry reveals active flight in *Archaeopteryx*" by Voeten et al.

Includes:
- 10 Supplementary Figures
- 3 Supplementary Tables
- 4 Supplementary Notes
- 35 Supplementary References

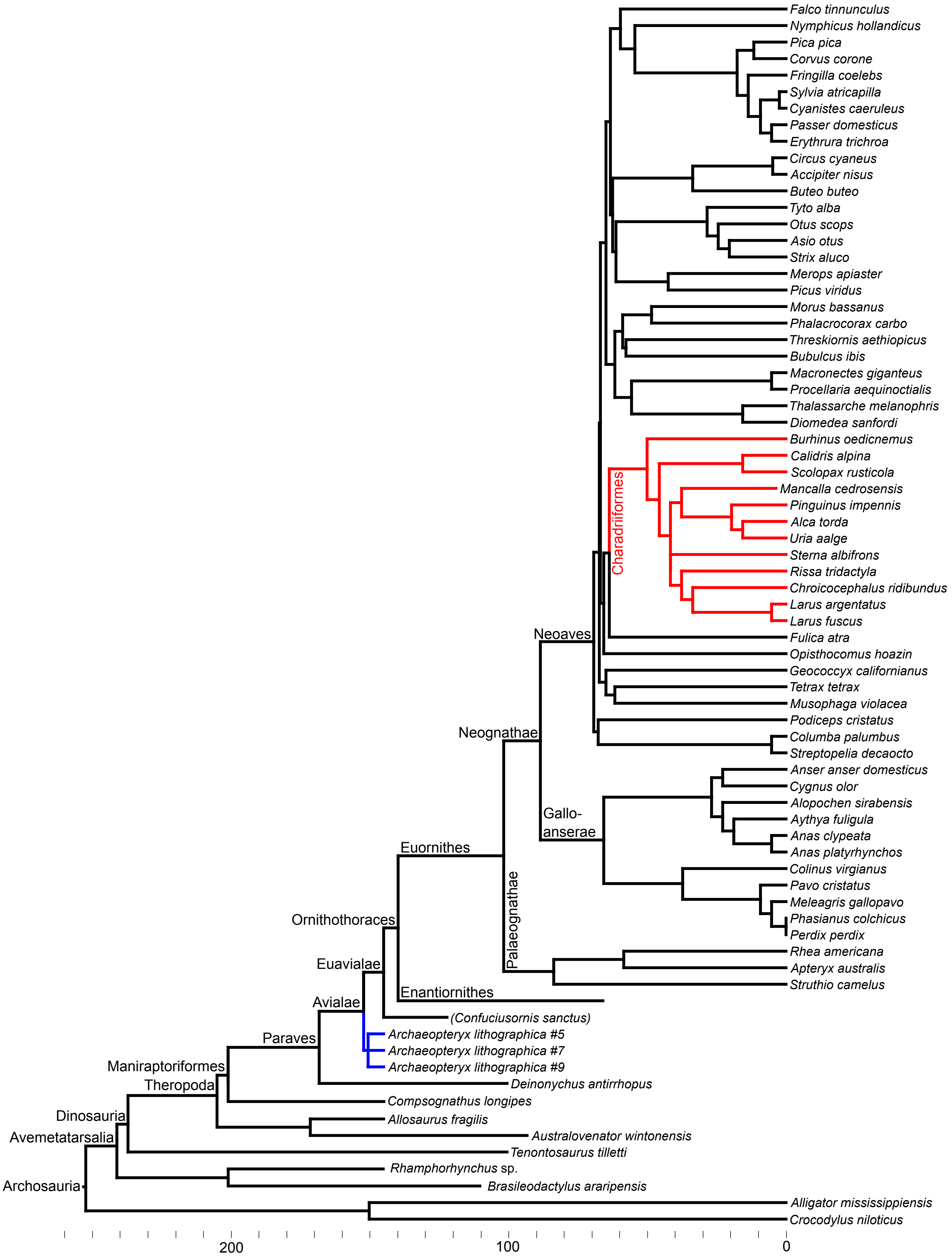

Supplementary Figure 1. Phylogenetic tree used in this study. X-axis in million years before present. Selected ranks specified. *Archaeopteryx* (blue) and Charadriiformes (red) indicated, *Confuciusornis sanctus* (between parentheses) not included in analysis. For sources see Supplementary Data 4.

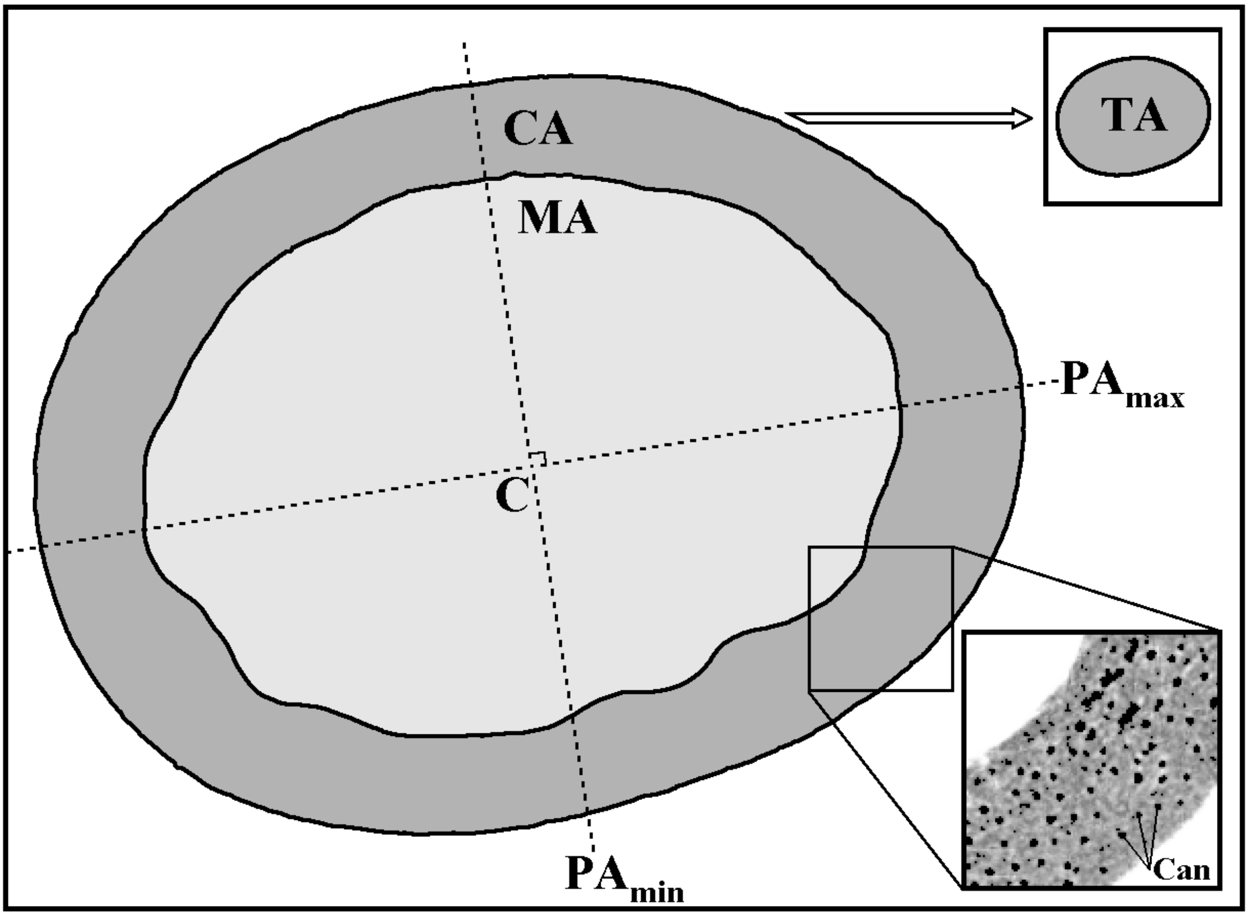

Supplementary Figure 2. Bone cross-sectional parameters. Schematic cross section of the pheasant humerus illustrating parameters and orientations obtained with ImageJ and MomentMacro for ImageJ: Cortical area (CA), Medullary area (MA), Total Area contained within periosteal margin (TA), Longest principal axis (PA_{max}), Shortest principal axis (PA_{min}), Section centroid (C), Cortical canals (Can indicates selected canals; accentuated here through ImageJ threshold selection). CA/TA, our index of relative cortical thickness, equals CA/(CA+MA). Polar moment of area J quantifies the resistance against torsion around the longitudinal bone axis (perpendicular to this section) and equals Imax+Imin; the second moments of area in the directions of PA_{max} and PA_{min}, respectively. In this study, J is normalised through division by body mass M. Cortical vascular density is defined as Can/CA and quantifies the average amount of canals present per mm^2 of cortical bone in cross section.

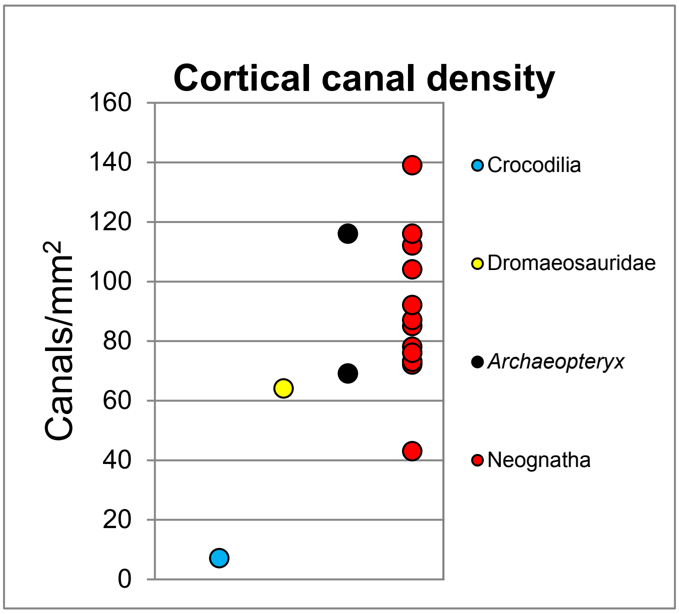

Supplementary Figure 3. Average cortical canal density across selected archosaurs. Cortical canal density of species expressed as CAN/mm^2. Values averaged over humerus and ulna, cortical canal density of Dromaeosauridae represents ulnar value exclusively. *Archaeopteryx* specimens plotted individually.

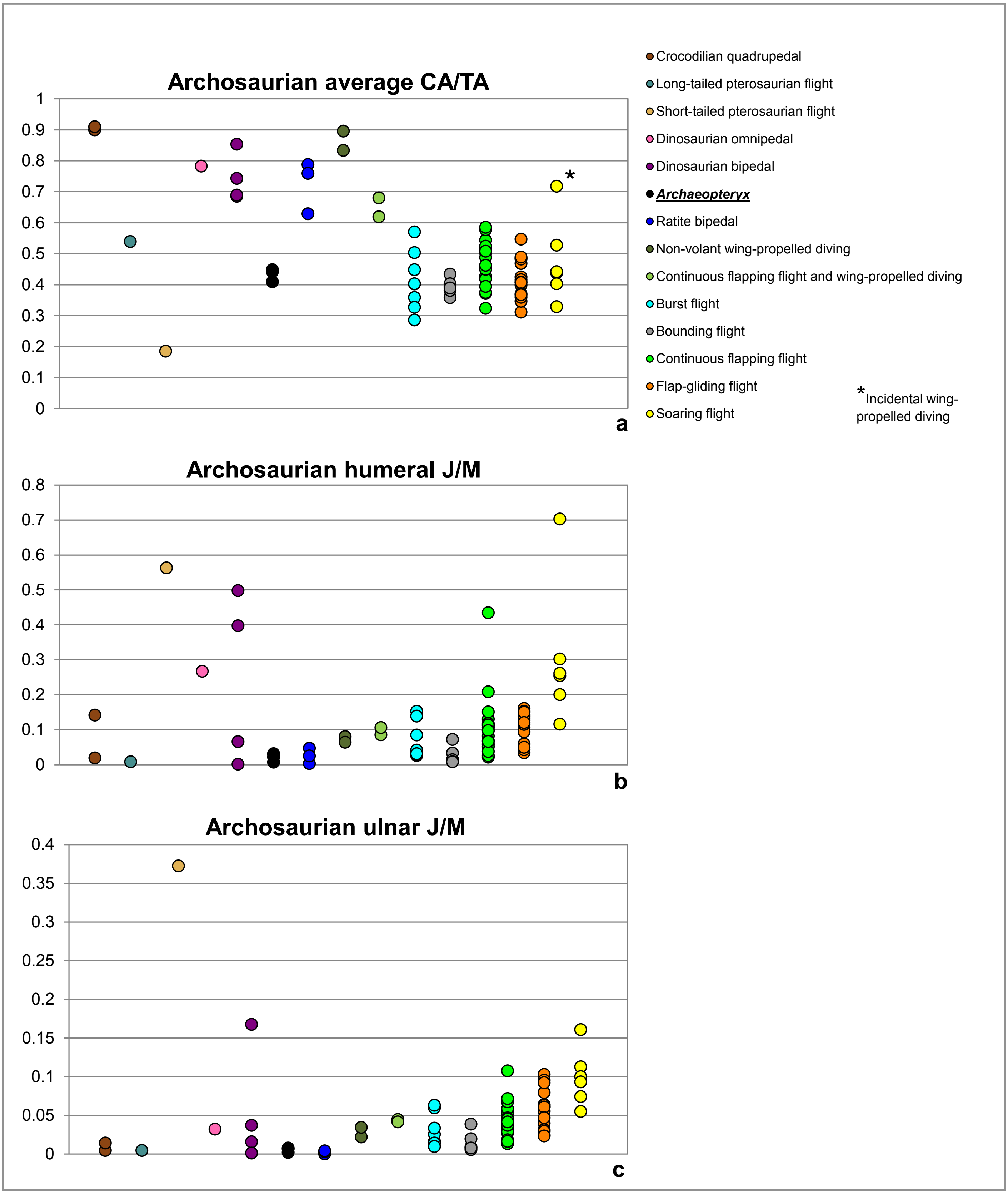

Supplementary Figure 4. Univariate plots of cross-sectional parameters of archosaurian anterior limb bones according to the locomotor classification modified after Close et al.[2]. a, Averaged humeroulnar relative cortical thickness. **b,** Mass-normalised humeral torsional resistance. **c,** Mass-normalised ulnar torsional resistance.

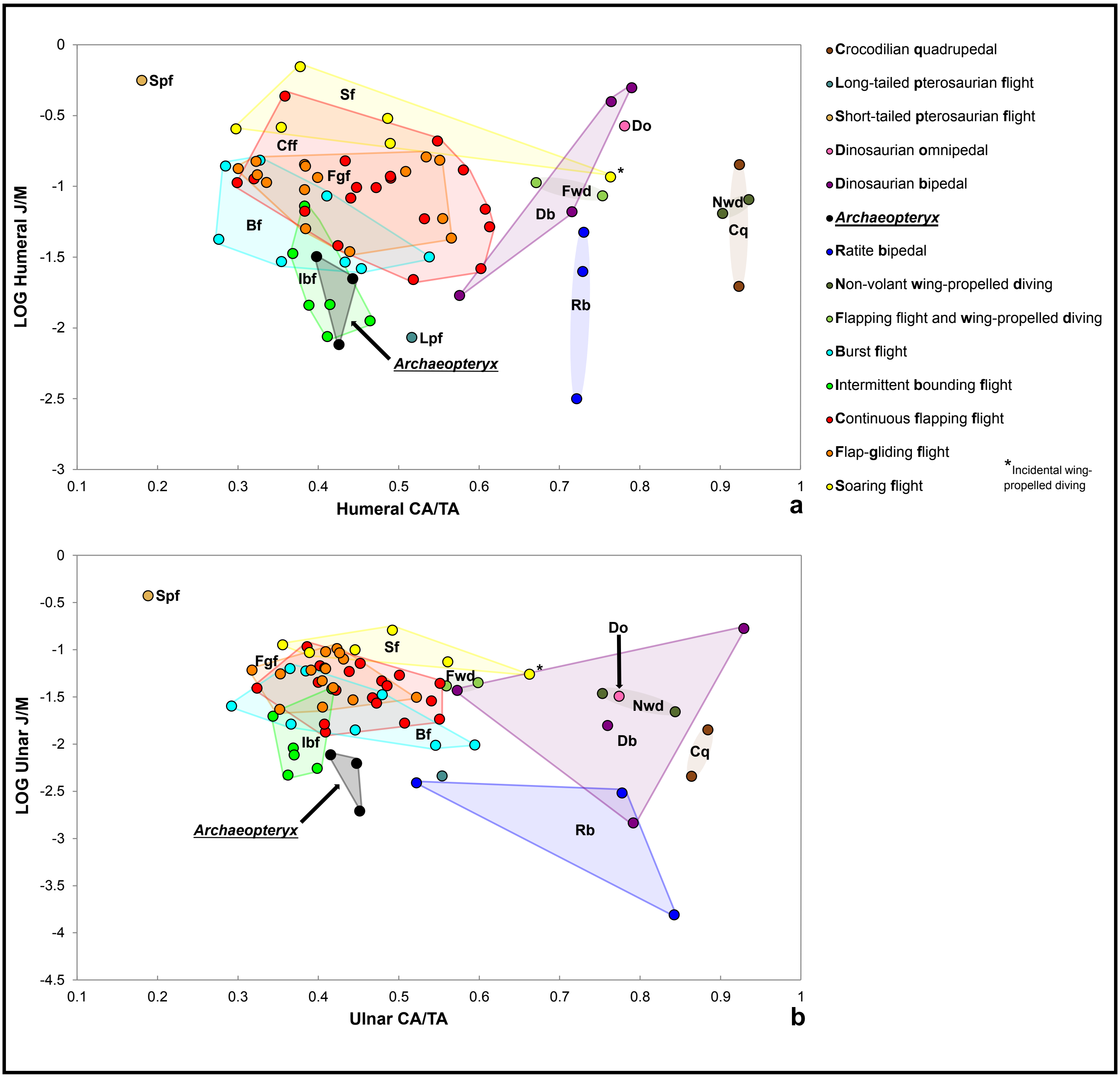

Supplementary Figure 5. Bivariate plots of cross-sectional parameters from archosaurian anterior limb bones according to the locomotor classification modified after Close et al.[2]. a, Humeral relative cortical thickness versus mass-normalised torsional resistance. **b,** Ulnar relative cortical thickness versus mass-normalised torsional resistance.

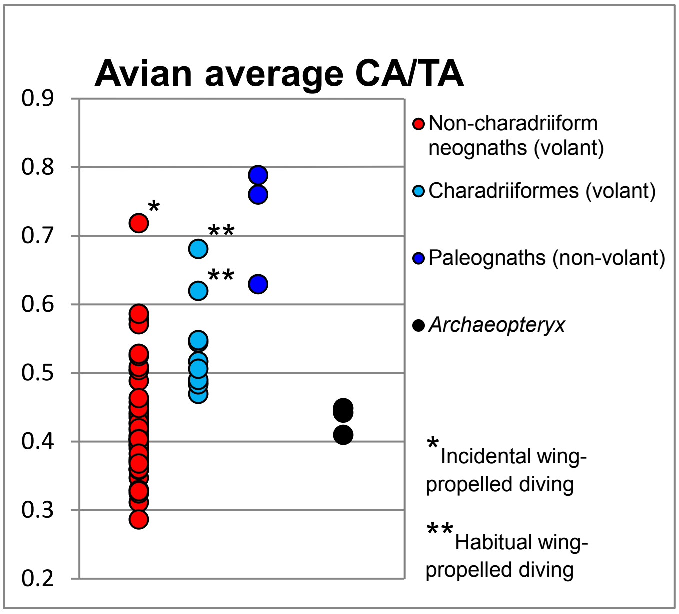

Supplementary Figure 6. Average relative cortical thickness across birds. Note the elevated relative cortical thickness of Charadriiformes with respect to non-charadriiform neognaths.

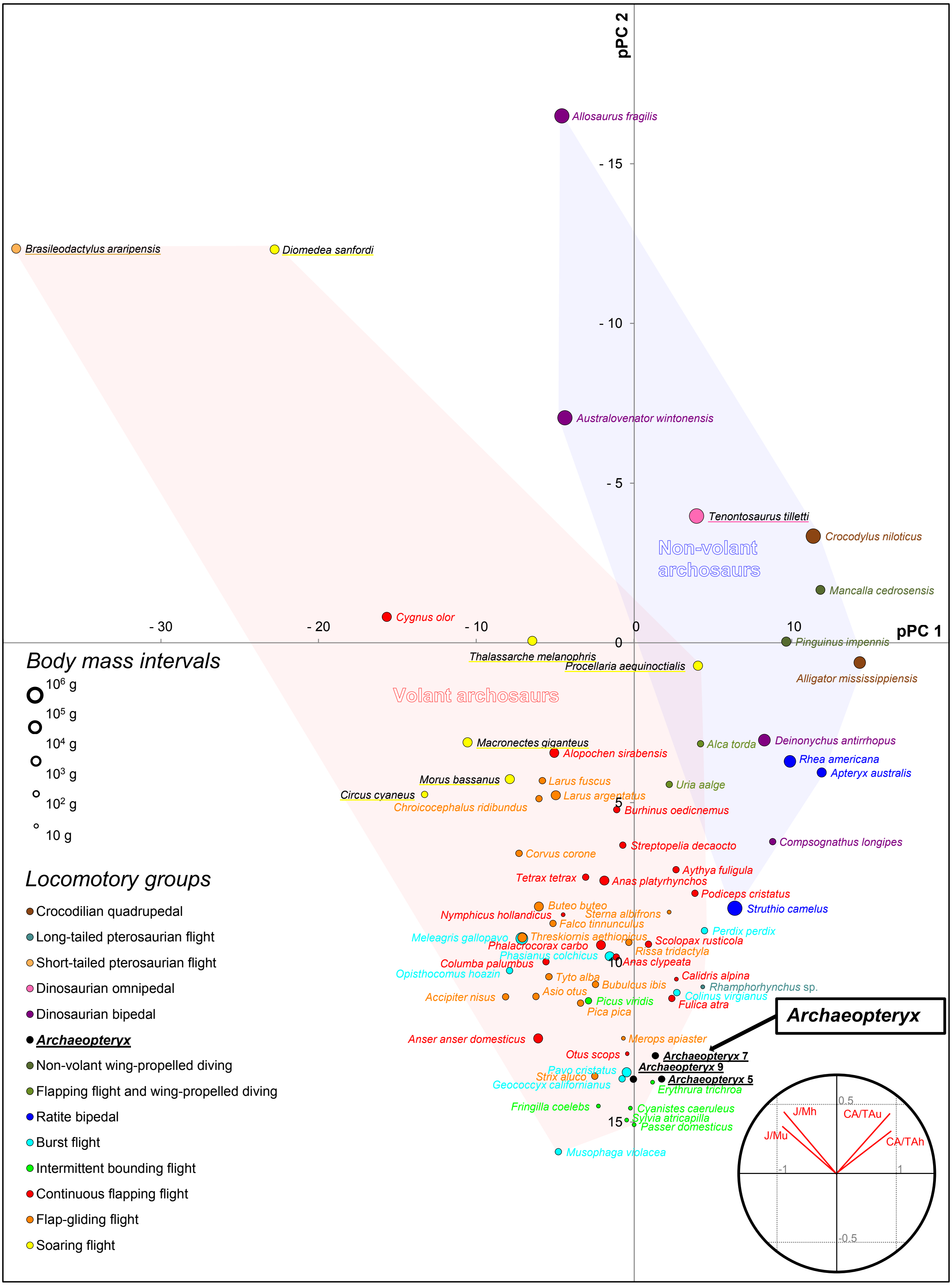

Supplementary Figure 7. First and second phylogenetic principal component plot for specific archosaurian humeral and ulnar relative cortical thickness (CA/TA) and mass-corrected polar moment of inertia of an area (J/M). Circles represent species, *Archaeopteryx* specimens plotted individually. Circle diameter reflects body mass class, circle color indicates locomotory strategy (adapted from the division by Close et al.[2]). Parameters labeled ¨_h¨ and ¨_u¨ in the loading biplot indicate humeral and ulnar affinity, respectively.

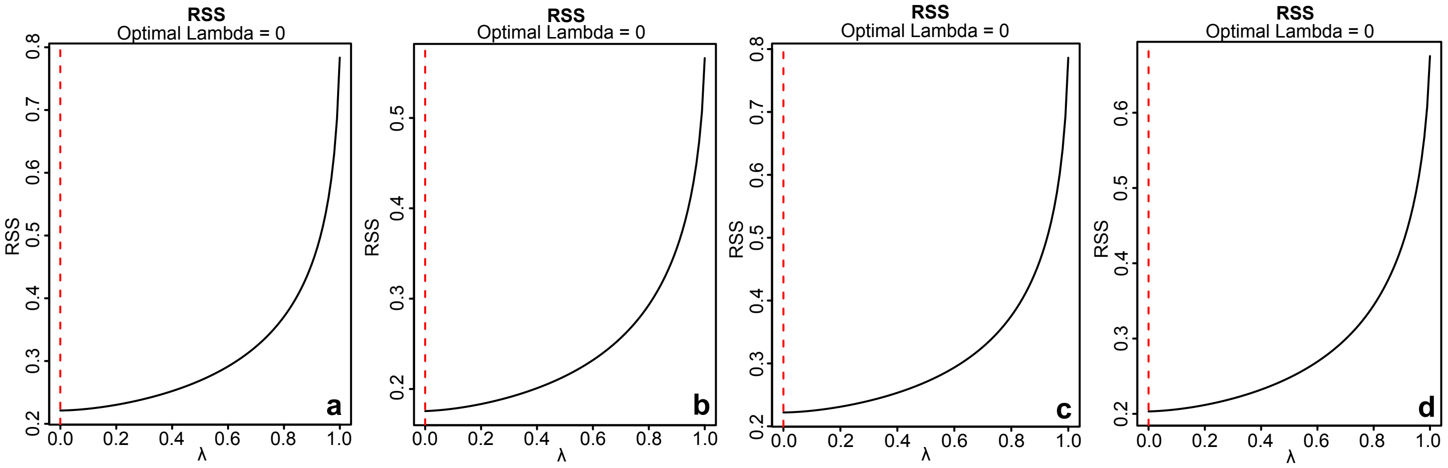

Supplementary Figure 8. Log-likelihood plots showing optimum value of Pagel's λ required to control for phylogenetic non-independence in phylogenetic Flexible Discriminant Analysis (pFDA). Phylogenetic independence is revealed for the data sets categorised following the locomotory divisions adapted from **a**, Viscor et al.[1], training taxa only, **b**, Viscor et al.[1], all taxa, **c**, Close et al.[2], training taxa only, **d**, Close et al.[2], all taxa.

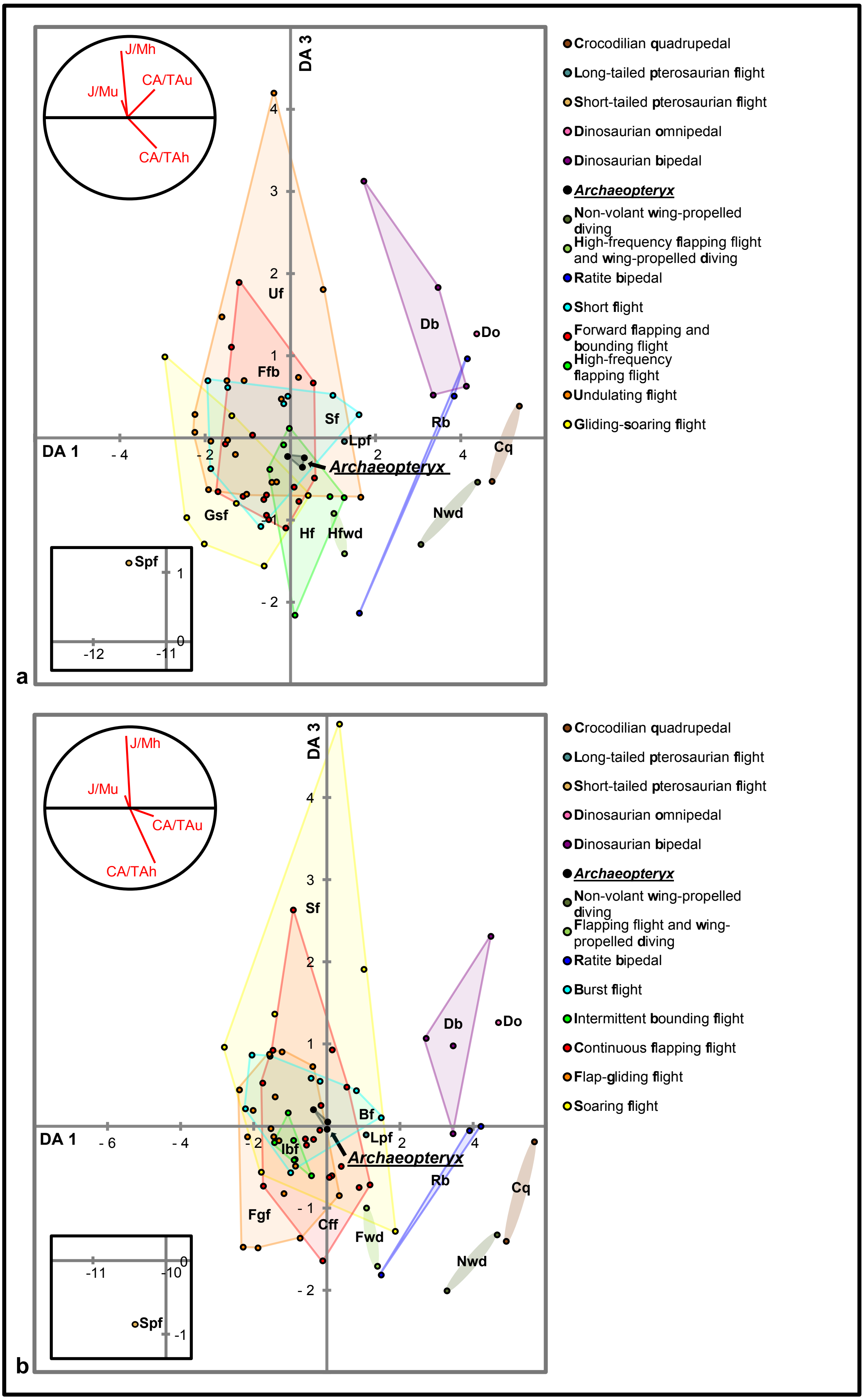

Supplementary Figure 9. LDA plot for specific archosaurian humeral and ulnar CA/TA and J/M. First and third linear discriminant axes are presented. Classification follows the locomotory divisions adapted from **a**, Viscor et al.[1] and **b**, Close et al.[2], non-pterosaurian flight strategies represent avian flight modes. Dots correspond to species, *Archaeopteryx* specimens plotted individually. Colored hulls delimit groups with a minimum of three representatives. Parameters labeled ¨_h¨ and ¨_u¨ in loading biplots designate humeral and ulnar affinity, respectively.

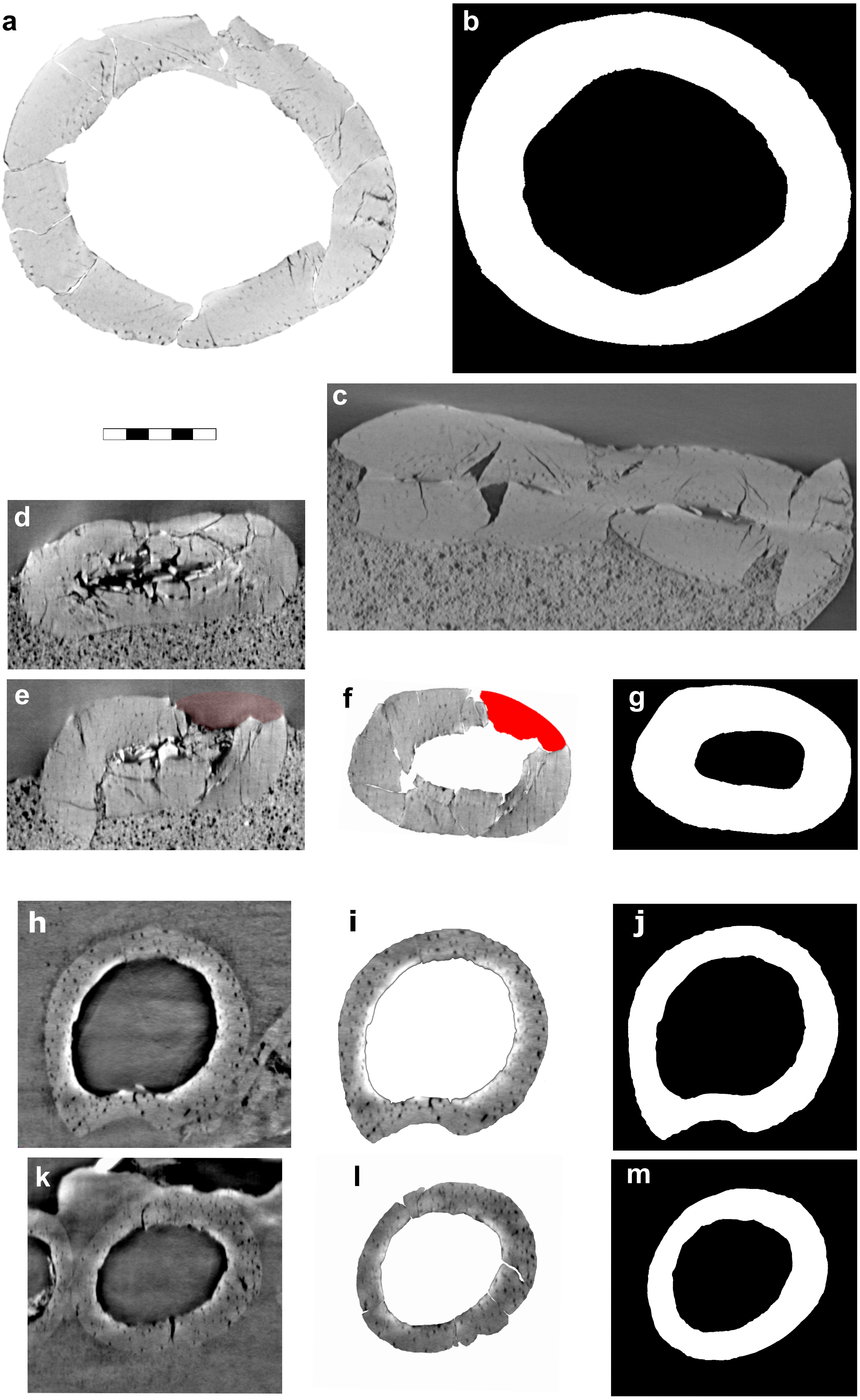

Supplementary Figure 10. *Compsognathus* and *Rhamphorhynchus* sectional data used in this study. a-c, *Compsognathus* left humeral **a,** restored section, **b,** binary section, and **c,** sampled virtual cross section. **d-g,** *Compsognathus* right ulnar **d,** virtual cross section 3.58 mm proximal to sampled location (e); note fractured cortex top right in image, **e,** sampled virtual cross section with supplemented cortical element top right in image, **f,** restored section, and **g,** binary section. **h-j,** *Rhamphorhynchus* right humeral **h,** sampled virtual cross section, **i,** restored section, and **j,** binary section. **k-m,** *Rhamphorhynchus* left ulnar **k,** sampled virtual cross section, **l,** restored section, and **m,** binary section. Scale bar measures 1 mm.

Supplementary Table 1. P-values with uncorrected significance of pairwise MANOVA including CA/TA_h, CA/TA_u, J/M_h and J/M_u for the classification adapted from Viscor et al.[1]. WPD designates wing-propelled diving. Mystery taxa and groups represented by a single specimen are not included. Red indicates failed tests, light red indicates insignificant difference in means, yellow indicates marginally significant difference in means (significant at CI=90%, insignificant at CI=95%), blue indicates significant difference in means (CI=95%).

**Locomotor mode (1)**	Crocodilian quadrupedal	Dinosaurian bipedal	Ratite bipedal	Non-volant (WPD)	Short Flight	Forward flapping/ bounding flight	High-frequency flapping flight	High-frequency flapping (WPD)	Undulating flight	Gliding/ Soaring flight
Crocodilian quadrupedal		0.73287	fail	fail	0.011561	0.00015631	0.10411	fail	4.66E-05	0.023769
Dinosaurian bipedal	0.73287		0.66948	0.67827	0.0049269	8.72E-05	0.047207	0.63156	4.66E-05	0.0062851
Ratite bipedal	fail	0.66948		fail	0.023877	0.0012843	0.16997	fail	0.0003783	0.025003
Non-volant (WPD)	fail	0.67827	fail		0.015474	0.00041253	0.15331	fail	0.00014284	0.043361
Short Flight	0.011561	0.0049269	0.023877	0.015474		0.52778	0.10757	0.092063	0.18763	0.017928
Forward flapping/ bounding flight	0.00015631	8.72E-05	0.0012843	0.00041253	0.52778		0.19453	0.033387	0.29029	0.021149
High-frequency flapping flight	0.10411	0.047207	0.16997	0.15331	0.10757	0.19453		0.65051	0.064075	0.17321
High-frequency flapping (WPD)	fail	0.63156	fail	fail	0.092063	0.033387	0.65051		0.018948	0.29628
Undulating flight	4.66E-05	4.66E-05	0.0003783	0.00014284	0.18763	0.29029	0.064075	0.018948		0.033829
Gliding/ Soaring flight	0.023769	0.0062851	0.025003	0.043361	0.017928	0.021149	0.17321	0.29628	0.033829	

Supplementary Table 2. P-values with uncorrected significance of pairwise MANOVA including CA/TA_h, CA/TA_u, J/M_h and J/M_u for the classification adapted from Close et al.[2]. WPD designates wing-propelled diving. Mystery taxa and groups represented by a single specimen are not included. Red indicates failed tests, light red indicates insignificant difference in means, yellow indicates marginally significant difference in means (significant at CI=90%, insignificant at CI=95%), blue indicates significant difference in means (CI=95%).

**Locomotor mode (2)**	Crocodilian quadrupedal	Dinosaurian bipedal	Ratite bipedal	Non-volant (WPD)	Burst flight	Bounding flight	Continuous flapping flight	Continuous flapping (WPD)	Flap-gliding flight	Soaring flight
Crocodilian quadrupedal		0.75026	fail	fail	0.01033	0.04736	0.00012138	fail	3.85E-05	0.070081
Dinosaurian bipedal	0.75026		0.55749	0.71276	0.0025153	0.0074973	4.44E-05	0.61262	7.19E-06	0.035217
Ratite bipedal	fail	0.55749		fail	0.023133	0.054083	0.0022136	fail	0.00027326	0.063626
Non-volant (WPD)	fail	0.71276	fail		0.015813	0.063726	0.00056317	fail	0.00017818	0.10014
Burst flight	0.01033	0.0025153	0.023133	0.015813		0.56165	0.1463	0.10972	0.076519	0.016082
Bounding flight	0.04736	0.0074973	0.054083	0.063726	0.56165		0.08788	0.22614	0.091914	0.016204
Continuous flapping flight	0.00012138	4.44E-05	0.0022136	0.00056317	0.1463	0.08788		0.11103	0.067407	0.01168
Continuous flapping (WPD)	fail	0.61262	fail	fail	0.10972	0.22614	0.11103		0.031402	0.32431
Flap-gliding flight	3.85E-05	7.19E-06	0.00027326	0.00017818	0.076519	0.091914	0.067407	0.031402		0.0059127
Soaring flight	0.070081	0.035217	0.063626	0.10014	0.016082	0.016204	0.01168	0.32431	0.0059127	

Supplementary Table 3. Statistics of one-way MANOVA of parameter set including CA/TA_h, CA/TA_u, J/M_h and J/M_u for the classifications adapted from Viscor et al.[1] and Close et al.[2]. Mystery taxa and groups represented by a single specimen not included.

**Viscor et al.[1]**			
Wilks´ lambda:	0.08448	Pillai trace:	1.492
df1:	36	df1:	36
df2:	200.4	df2:	224
F:	5.196	F:	3.701
p (same):	1.29E-14	p (same):	9.72E-10

**Close et al.[2]**			
Wilks´ lambda:	0.06962	Pillai trace:	1.59
df1:	36	df1:	36
df2:	200.4	df2:	224
F:	5.766	F:	4.105
p (same):	1.75E-16	p (same):	3.19-11

Supplementary Notes

Supplementary Note 1: Accumulation mode for tomographic acquisition

X-ray tomography relies on the image contrast present in an X-ray beam after transmission through an object. It therefore requires the detection of a signal that is significantly different from both the incoming beam and the electronic noise of the detector. However, an object absorbing most of the incoming signal during imaging may result in recorded data statistically indiscriminable from the electronic noise of the detector. To negotiate this effect, two solutions are available: 1) increasing the energy (and thus the penetration) of the incoming beam, or 2) extended sampling of the incoming signal by prolonging the exposure time. The energy range available in an X-ray tomographic setup depends on inherent properties of the source (e.g., the maximum voltage for laboratory X-ray tomographs, or electron beam energy in the storage ring and the magnetic field in insertion devices or bending magnets for synchrotron radiation). Such a setup has a maximum operational energy level threshold that often cannot be exceeded. Furthermore, since transmission increases with energy, raising the operational energy level may adversely affect the relative contrast of interest in the tomographic reconstruction. It is therefore often preferable to amplify the recorded signal by prolonging the exposure time. Most X-ray tomographic setups record data in 16 bit, which corresponds to a dynamic range of 65535 grey levels. The exposure time is ideally set to approach the saturation limit in the flat-field image as closely as possible without actually achieving saturation. However, the exposure time can only be prolonged to a certain maximum. In specific cases, traditional setups do not allow for a sufficiently long exposure time without saturation of the detector.

A designated setup was developed at the ESRF to circumvent this problem[33,34]. Implementation of the so-called attenuation protocol involves the application of three physical components in the tomographic setup. Firstly, a cylinder with a diameter equal to or slightly larger than the observed horizontal field of view is filled with a material that has a

density close to that of the sample medium of interest (e.g. small glass or aluminium micro balls when associated with fossilised remains). Secondly, a solid U-shaped block, the profiler, is installed to neutralise the laterally variable absorption of the cylinder. The specific geometry of the profiler induces a stronger absorption laterally than centrally, which normalises the signal. Thirdly, a solid and semicircular block composed of the same material as the profiler, the attenuator, is applied to achieve homogenised absorption during flat-field correction. When the sample is placed in the cylinder filled with micro balls during tomographic acquisition, the exposure time can be sufficiently prolonged to obtain and record an adequately detectable signal while preventing saturation of the object edges. During recording of the flat-field images (incoming beam without interference), the profiler and attenuator are placed in the beam to emulate the acquisition configuration of profiler plus cylinder with micro balls. The subsequent flat-field correction thus acts principally on the contrast induced by the sample.

Although the attenuation protocol enabled experiments that were previously impossible[33,35], the associated setup does introduce two new problems. Firstly, perfect alignment of all the components in the optic path is crucial but proved challenging and tedious. Secondly, and more importantly, containing a fossil in a cylinder filled with glass or aluminium micro balls is often impractical and may damage delicate structures. Imaging a fossil preserved on a lithic slab was particularly challenging, since immersing it completely in micro balls would result in an exceedingly heavy setup. The alternative of placing the fossil in a smaller cylinder with vertical slots that allow the slab to extend beyond the cylinder itself is problematic in that achieving a sufficiently good seal between the sample and the cylinder to prevent micro balls from escaping without risking damage to the fossil itself poses a substantial challenge.

The PCO.edge 4.2 and PCO.edge 5.5 (PCO AG, Kelheim, Germany) have gradually replaced the ESRF proprietary FReLoN-2K as the preferred detectors, largely because of their higher recording frame rate achieved through a lower full-well capacity (each pixel of

the PCO.edge detectors is saturated at an approximately ten-fold lower electron count than the FReLoN-2k). Although both detector families share a dynamical range of 14 bit, the signal-to-noise ratio of the PCO.edge detectors is inherently inferior to that of the FReLoN-2k, which was initially overcome by increasing the amount of projections per scan. Although this does increase overall X-ray sampling per rotation of the object, it also increases tomographic reconstruction times significantly.

To overcome particular shortcomings of the attenuation protocol, we developed the accumulation mode that exploits the high recording frame rate of the PCO.edge detectors by essentially summing multiple images to produce a single image. As in traditional acquisition, a projection is generated over a given angular range during the rotation of the object. However, in accumulation mode and with the accumulation count set to 10, the camera records images every 10th of that angular range and transmits the frames to a device server where those 10 images are progressively summed to generate a single image. Because the dynamic range of the resulting images can exceed the 65535 grey values of 16-bit images, the projection is recorded as a 32-bit image. Doubling the bit depth also doubles the file size of the final image, but the amount of accumulated images prior recording does not further impact data size. Consequently, where doubling the number of projections has the same effect as applying an accumulation count of 2, an accumulation count of 10 is equivalent to a ten-fold increase in the amount of projections being recorded for only twice the occupied disk space. This approach is similar to the “average mode” available on selected laboratory tomographs. Compared to the attenuation protocol used before, the accumulation mode is vastly simpler to implement. While the attenuation protocol increases image dynamic in the most strongly absorbing parts of the sample, accumulation mode only offers improved sampling of the X-ray signal itself. As such, the accumulation mode does not solve the challenge of complete attenuations, but does provide a broader dynamic range for specifically fossils on lithic slabs oriented perpendicularly to the X-ray beam. Conversely, the differential transmission within the slab and at its surface reinforces the diffusion in the

scintillator when the width of the slab is oriented parallel to the X-ray beam, which results in an artificial lightening of grey levels at the surface of the slab. Although this effect does cause artefacts along the surface of the slab (e.g. Fig. 1 e and 1 f) that may interfere with subsequent analyses, the accumulation mode is to be preferred over the attenuation protocol when imaging potentially fragile specimens, as it prevents physical contact with the fossil itself.

Supplementary Note 2: Functional interpretation

Although CA/TA values for *Archaeopteryx* were found to exclusively fall within the range occupied by modern volant birds, J/M values of particularly the smallest (fifth) specimen of *Archaeopteryx* plot within those of small (body mass < 26g) extant volant birds and flightless archosaurs. There are three reasons why we believe this transitional signal is more consistent with early avian volancy than with (retained) non-volancy. Firstly, high CA/TA values account for the generalised non-volant archosaurian condition, including the ancestral pre-avian condition (>95%). The departure of *Archaeopteryx* from this condition into the range of exclusively volant birds represents a functional adaptation that is most parsimoniously explained by selective pressures relating specifically to volancy. Secondly, the secondarily flightless paleognaths record a return to CA/TA values in the range of non-volant archosaurs and few wing-propelled diving birds, which indicates the presence of sufficient selective pressure to induce cortical thickening when the demands of volancy are relinquished. Thirdly, a decrease in CA/TA implies less bone mass is present within a given periosteal margin. All other factors being equal, this will inherently result in an absolute decrease in J, which we observe is compensated for particularly in modern highly aerial flap-gliding and soaring birds, but also in the trajectory from long- to short-tailed pterosaurs, by a redistribution of bone mass.

Additionally, the reconstructed ulnar geometry of the seventh specimen (Fig. 1 l) of *Archaeopteryx* suffers from a poorly resolved cortical interval in the original data (most left in

Fig. 1 f) that describes a suspicious cortical curvature. We choose not to correct for this structural artefact as to not introduce irreproducible manipulation. Since this artefact results in a reconstructed bone geometry presumably less circular than the original *in vivo* condition, the recovered value for J likely represents an underestimation.

Supplementary Note 3: Phylogenetic considerations towards tree topology and timing

Two particular inconsistencies emerged during creation of the chronogram (see Supplementary Figure 1 and Supplementary Data 3) through PaleoDB[27]. Firstly, *Chroicocephalus ridibundus* is lacking from PaleoDB, yet *Larus ridibundus* is present, albeit without associated specimens. Here, we followed Pons et al.[31] in recognition of the genus *Chroicocephalus*. Secondly, the oldest record of the genus *Phasianius* in PaleoDB was recognised as an erroneous entry; this in fact considers the grouse *Archaeophasianus mioceanus*[8].

Supplementary Note 4: Statistical analyses

Phylogenetic univariate analysis revealed that both humeral and ulnar CA/TA offer statistically significant discrimination between several archosaurian locomotor modes in our data set (Supplementary Table 1) for both locomotor classifications tested (Supplementary Data 4). Specific average CA/TA values below 0.60 are only present in volant forms, whereas non-volant archosaurs exclusively exhibit specific average CA/TA values over 0.60. Wing-propelled diving in volant birds is occasionally associated with average CA/TA values in the range of non-volant archosaurs. *Archaeopteryx* exhibits low average CA/TA values that range between 0.40 and 0.46. Humeral J/M alone does not contribute significantly to locomotor discrimination (Supplementary Data 5) and lacks a significant size effect (Supplementary Data 5). As such, humeral J/M supplements the functional distinction of ulnar J/M, which does provide significant separation but also retains a small yet significant residual size effect (Supplementary Data 5). Multivariate analyses thus involved humeral and ulnar CA/TA and J/M to elucidate on wing function in *Archaeopteryx*.

The first three phylogenetic principal components extracted through phylogenetic PCA of the entire data set (Supplementary Data 2) explain 79.99%, 16.07% and 2.67% of total variance. Partitioning Around Medoids of these three phylogenetic principal components set to two clusters (Supplementary Data 2) explains 78.27% of total point variability. The two recovered clusters were found to primarily recapitulate the separation between known volant and non-volant archosaurian taxa. Only the (incidental) wing-propelled diving birds *Alca torda*, *Procellaria aequinoctialis* and *Uria aalge* (4.54% of training taxa set) incorrectly group with non-volant archosaurs.

Contrasting pPCA scores with (non-phylogenetic) LDA results revealed identical group assignments that underline the phylogenetic independence of the traits considered. Post-hoc pairwise MANOVA confirmed a significant relation between locomotory strategy and the parameter set used (Supplementary Table 3). The resolved affinity of *Archaeopteryx* with the short[1] and burst[2] flight categories (Supplementary Data 2) is reinforced by the observation that volant birds employing other flight strategies yet recovered close to both *Archaeopteryx* and the short[1]/burst[2] flyers in discriminant (and pPCA) morphospace typically exhibit a markedly lower body mass (Supplementary Data 1 and Supplementary Fig. 7).

Supplementary References

1. Viscor, G. & Fuster, J. F. Relationships between morphological parameters in birds with different flying habits. *Comp. Biochem. Physiol.* **87A,** 231-249 (1987).
2. Close, R. A. & Rayfield, E. J. Functional morphometric analysis of the furcula in Mesozoic birds. *PloS one* **7,** e36664 (2012).
3. De Margerie, E., Sanchez, S., Cubo, J. & Castanet, J. Torsional resistance as a principal component of the structural design of long bones: Comparative multivariate evidence in birds. *The Anatomical Record Part A: Discoveries in Molecular, Cellular, and Evolutionary Biology* (2005).
4. Cubo, J., & Casinos, A. Biomechanical significance of cross-sectional geometry of avian long bones. *European Journal of Morphology* **36**, 19-28 (1998).
5. Smith, N. A. & Clarke, J. A. Osteological Histology of the Pan-Alcidae (Aves, Charadriiformes): Correlates of Wing-Propelled Diving and Flightlessness: Osteological Histology of The Pan-Alcidae. *The Anatomical Record* **297,** 188–199 (2014).
6. Bybee, P. J., Lee, A. H., & Lamm, E. T. Sizing the Jurassic theropod dinosaur Allosaurus: assessing growth strategy and evolution of ontogenetic scaling of limbs. *Journal of Morphology* **267,** 347-359 (2006).
7. Parsons, W. L. & Parsons, K. M. Further descriptions of the osteology of Deinonychus antirrhopus (Saurischia, Theropoda). *Bulletin of the Buffalo Society of Natural Sciences* **38,** 43-54 (2009).
8. White, M. A., Cook, A. G., Hocknull, S. A., Sloan, T., Sinapius, G. H., & Elliott, D. A. New forearm elements discovered of holotype specimen Australovenator wintonensis from Winton, Queensland, Australia. *PloS One* **7,** e39364 (2012).
9. Werning, S. The ontogenetic osteohistology of Tenontosaurus tilletti. *PLoS One* **7,** e33539 (2012).

10. Woodward, H. N., Horner, J. R. & Farlow, J. O. Quantification of intraskeletal histovariability in Alligator mississippiensis and implications for vertebrate osteohistology. *PeerJ* **2,** e422 (2014).
11. De Magalhaes, J. P., & Costa, J. A database of vertebrate longevity records and their relation to other life-history traits. *Journal of evolutionary biology* **22,** 1770-1774 (2009). Retrieved from on http://genomics.senescence.info/species/ on 18 January 2016.
12. Cubo, J. & Casinos, A. Incidence and mechanical significance of pneumatization in the long bones of birds. *Zoological Journal of the Linnean Society* **130,** 499–510 (2000).
13. Andrews, C. On some fossil remains of carinate birds from Central Madagascar. *Ibis* **39,** 343-359 (1897).
14. Livezey, B. C. Morphometrics of flightlessness in the Alcidae. *The Auk* **105,** 681-698 (1988).
15. del Hoyo, J., Elliott, A., Sargatal, J., Christie, D. A., & de Juana, E. *Handbook of the Birds of the World Alive* (Lynx Editions, 2015). Retrieved from http://www.hbw.com on 19 January 2016.
16. Macky, R. & White, B. (eds.) *Gastrointestinal Microbiology: Volume 1 Gastrointestinal Ecosystems and Fermentations* (Nimrod Book Services, 1996).
17. Erickson, G. M. *et al.* Was Dinosaurian Physiology Inherited by Birds? Reconciling Slow Growth in Archaeopteryx. *PLoS ONE* **4,** e7390 (2009).
18. Paul, G. S. *Predatory dinosaurs of the world: a complete illustrated guide* (Simon & Schuster, 1988).
19. Paul, G. S. *The Princeton field guide to dinosaurs* (Princeton University Press, 2010).
20. Kellner, A. W. A., & Tomida, Y. Description of a new species of Anhangueridae (Pterodactyloidea) with comments on the pterosaur fauna from the Santana Formation (Aptian-Albian), northeastern Brazil. *National Science Museum Monographs* **17,** 1-135 (2000).

21. Veldmeijer, A. J. Pterosaurs from the Lower Cretaceous of Brazil in the Stuttgart Collection. *Stuttgarter Beitr. Naturk. Ser. B.* **327,** 1-27 (2002).
22. Wellnhofer, P. Weitere Pterosaurierfunde aus der Santana-Formation (Apt) der Chapada do Araripe, Brasilien. *Palaeontographica Abteilung A.* **187,** 43-101 (1991).
23. Henderson, D. M. Pterosaur body mass estimates from three-dimensional mathematical slicing. *Journal of Vertebrate Paleontology* **30,** 768-785 (2010).
24. Witton, M. P. in *Special volume: Flugsaurier: pterosaur papers in honour of Peter Wellnhofer* (Buffetaut, E. & Hone, D. W. E. eds.) 143-158 (München, 2008).
25. Masser, M. P. Alligator Production: Grow-out and harvest. *SRAC Publication* **232**, 1-4 (1993).
26. Huchzermeyer, F. W. *Crocodiles: Biology, Husbandry, and Diseases* (CABI, 2003).
27. Paleobiology Database (2016). Retrieved from http://paleobiodb.org on 29 January 2016.
28. Jarvis, E. D. *et al.* Whole-genome analyses resolve early branches in the tree of life of modern birds. *Science* **346**, 1320-1331 (2014).
29. Bengtson, S.-A. Breeding ecology and extinction of the great auk (Pinguinus impennis): anecdotal evidence and conjectures. *The Auk* **101,** 1-12 (1984).
30. Turvey, S. T. (ed.) *Holocene extinctions*. (OUP Oxford, 2009).
31. Pons, J.-M., Hassanin, A., Crochet, P.-A. Phylogenetic relationships within the Laridae (Charadriiformes: Aves) inferred from mitochondrial markers. *Molecular Phylogenetics and Evolution* **37,** 686-699 (2005).
32. Johnsgard, P.A. *Grouse and quails of North America* (University of Nebraska Press, 1973).
33. Carlson, K. J., Stout, D., Jashashvili, T., de Ruiter, D. J., Tafforeau, P., Carlson, K. & Berger. L. R. The endocast of MH1, *Australopithecus sediba*. *Science* **333**, 1402-1407 (2011).

34. Sanchez, S., Fernandez, V., Pierce, S. E. & Tafforeau, P. Homogenization of sample absorption for the imaging of large and dense fossils with synchrotron microtomography. *Nature Protocols* **8**, 1708–1717 (2013).

35. Fernandez, V., Abdala, F., Carlson, K. J., Collins Cook, D., Rubidge, B. S., Yates, A. & Tafforeau, P. Synchrotron reveals Early Triassic odd couple: injured amphibian and aestivating therapsid share burrow. *PLoS One* **8**, e64978 (2013).
