# Supplementary material for: Wing bone geometry reveals active flight in Archaeopteryx
Source: Nat Commun. 2018 Mar 13;9:923. doi: 10.1038/s41467-018-03296-8 (PMC5849612; doi:10.1038/s41467-018-03296-8)
Supplement: Supplementary file 3 — Description of Additional Supplementary Files [file 41467_2018_3296_MOESM3_ESM.pdf]

Description of Additional Supplementary Files

File Name: Supplementary Data 1
Description: Raw data and declaration of external data sources. Organised by taxon, includes raw data values, locomotor mode, and body mass.

File Name: Supplementary Data 2
Description: Multivariate statistics underlying this study. Includes raw pPCA scores, results of k-means clustering, PAM of pPCA scores, and LDA classification.

File Name: Supplementary Data 3
Description: Data acquisition parameters for synchrotron µCT. Scanning parameters as used on beamlines BM05 and ID19 of the European Synchrotron Radiation Facility.

File Name: Supplementary Data 4
Description: Motivation for topography and timing of chronogram. Organised by node, includes timing for both extinct and extant taxa and declares corresponding sources. Remarks provided where relevant.

File Name: Supplementary Data 5
Description: P-values for phylogenetic ANCOVA. Conducted using locomotory division as response variable, individual parameters as variates, and body mass as covariates. Non-phylogenetic p-values and f-values provided for reference.
